# The role and management of dietary and environmental factors in pediatric atopic dermatitis

**DOI:** 10.3389/fped.2026.1862285

**Published:** 2026-09-07

**Authors:** Xinlin Huang, Juan Ye, Liya Wu

**Affiliations:** 1 Department of Dermatology, Renmin Hospital, Hubei University of Medicine, Shiyan, Hubei, China; 2 Department of Nursing, First Affiliated Hospital, School of Medicine, Zhejiang University, Hangzhou, China

**Keywords:** dietary factors, environmental factors, intervention strategy, pediatric atopic dermatitis, quality of life, retrospective analysis

## Abstract

**Background:**

The pathogenesis of pediatric atopic dermatitis (AD) is complex, and the role of dietary and environmental factors in its onset and progression warrants systematic investigation.

**Objective:**

To explore the associations between dietary allergen sensitization, environmental factors, and the development of pediatric AD through a retrospective case-control analysis, and to evaluate the clinical efficacy of a multidimensional intervention strategy.

**Methods:**

This study employed a two-stage design. The first stage was a retrospective case-control study, enrolling 535 pediatric AD patients diagnosed between 2022 and 2024 and age- and sex-matched healthy children (*n* = 535). Conditional logistic regression models were used to analyze the associations between variables—including dietary allergen sensitization (milk/egg), air pollution index (PM2.5), indoor humidity, and skin microbiota characteristics (Staphylococcus aureus colonization rate)—and AD onset. The second stage was a prospective interventional study, enrolling 66 AD children who met the full inclusion criteria between January and December 2025. Participants were divided into an intervention group (*n* = 33, home-based care + allergen-specific dietary guidance) and a control group (*n* = 33, conventional intervention) according to the intervention modality. Propensity score matching (PSM) was applied to balance baseline characteristics. Differences in clinical efficacy, quality of life (CD-LQI score), sleep quality (PSQI score), and disease control (ADCT score) were compared between the two groups after three months of intervention, with additional analysis of changes from baseline to follow-up.

**Results:**

Conditional logistic regression analysis revealed that early milk/egg sensitization (AOR = 3.18, 95%CI: 2.08–4.78), winter indoor humidity <40% (AOR = 1.92, 95%CI: 1.30–2.71), PM2.5 >35 μg/m^3^ (AOR = 2.48, 95%CI: 1.80–3.50), and Staphylococcus aureus colonization rate >50% (AOR = 4.08, 95%CI: 2.98–5.58) significantly increased the risk of AD onset (all *P* < 0.05). The interventional study showed that the clinical efficacy rate in the intervention group was significantly higher than that in the control group (81.82% vs. 45.45%, *P* < 0.05). Furthermore, the intervention group demonstrated significantly better outcomes in CD-LQI score (9.01 ± 1.24 vs. 11.47 ± 1.52, *P* < 0.05), PSQI score (6.14 ± 1.21 vs. 8.23 ± 1.33, *P* < 0.05), and ADCT score (6.73 ± 1.54 vs. 12.58 ± 1.48, *P* < 0.05). The mean reductions (Δ) from baseline to three months in the intervention group were significantly greater than those in the control group for all three measures: CD-LQI (9.10 ± 2.01 vs. 6.85 ± 2.21, *P* < 0.001), PSQI (10.84 ± 3.12 vs. 8.66 ± 2.78, *P* < 0.001), and ADCT (11.50 ± 2.34 vs. 5.61 ± 2.21, *P* < 0.001).

**Conclusion:**

Dietary avoidance should be based on documented allergen sensitization and integrated with nutritional assessment, while environmental regulation should focus on humidity control and microbial homeostasis restoration. A multidimensional intervention strategy based on dietary and environmental factors (home-based care + allergen-specific dietary guidance) can effectively control pediatric AD, improve quality of life and sleep quality, and provide evidence-based support for the clinical management of AD.

## 1 Introduction

Atopic dermatitis (AD) is the most common chronic inflammatory skin disease in childhood, and its global prevalence has continued to rise in recent years. In China, epidemiological data indicate that the prevalence of AD among children aged 1–7 years has reached 12.94%, with a markedly higher prevalence in urban children than in their rural counterparts—a disparity suggesting that environmental factors likely play a significant role in disease occurrence. The clinical manifestations of AD extend far beyond impaired skin barrier function, intense pruritus, and recurrent eczematous lesions; owing to its protracted and relapsing course, the quality of life of affected children is severely compromised. Moreover, AD frequently co-occurs with other allergic conditions such as asthma and allergic rhinitis, forming the characteristic “atopic march” and imposing a substantial burden on families and healthcare systems (1–4).

Recent research has yielded deeper insights into the specific mechanisms through which dietary and environmental factors contribute to the pathogenesis ofAD. At the dietary level, common food allergens such as milk and egg can trigger immune responses either via IgE-mediated classical allergic pathways or through non-IgE-dependent cell-mediated immune routes, thereby inducing or exacerbating cutaneous inflammation. The timing and dosage of early allergen exposure may directly influence the establishment of immune tolerance. On the environmental front, air pollutants (such as fine particulate matter PM2.5) have been shown to activate oxidative stress signaling pathways, disrupt the normal expression of skin barrier-related proteins, and promote the proliferation of colonizing bacteria including Staphylococcus aureus, thereby intensifying local inflammatory states. Low indoor humidity during winter, on the other hand, compromises the integrity of the epidermal lipid barrier and increases transepidermal water loss, creating conditions favorable for disease onset (5–7). Additionally, dysbiosis of the skin microbiome, particularly excessive colonization by Staphylococcus aureus, has been repeatedly confirmed to correlate closely with AD severity and recurrence risk.

Despite the substantial body of evidence supporting the roles of the aforementioned factors, most existing studies have been confined to single-factor analyses, with a notable paucity of multidimensional investigations addressing the interactive effects between dietary and environmental factors. Equally noteworthy is the insufficient clinical evidence surrounding comprehensive intervention strategies such as “dietary avoidance combined with environmental regulation.” Against this backdrop, the present study first employs a retrospective case-control design to systematically analyze the associations between dietary allergen sensitization, air pollution exposure, indoor humidity conditions, skin microbiota characteristics, and the onset of pediatric AD. Second, through a clinical interventional study, it further evaluates the comprehensive effects of a multidimensional strategy—“allergen-specific dietary guidance + home-based care”—on clinical symptoms, quality of life, and sleep quality in affected children. We anticipate that this work will not only provide new perspectives for understanding the synergistic effects of dietary and environmental factors in the pathogenesis of pediatric AD but also accumulate scientific evidence to inform the development of individualized, multidimensional management strategies in clinical practice, thereby exerting a tangible impact on optimizing long-term disease management and improving the long-term prognosis of affected children.

## 2 Materials and methods

This study was approved by the Ethics Committee of the First Affiliated Hospital of Zhejiang University School of Medicine (approval number: 2021-032). The research was conducted in accordance with the Declaration of Helsinki and relevant ethical guidelines. For the retrospective case-control phase, which involved only the analysis of existing medical records and did not entail patient interaction or intervention, the requirement for informed consent was waived by the Ethics Committee. For the prospective interventional phase, written informed consent was obtained from all participants' parents or legal guardians prior to enrollment. Standardized operating procedures were established for data collection, and data entry was performed using a double-blind method with logical verification to ensure the scientific validity and reliability of the research findings.

### 2.1 Study design

The overall design of this study adopted a two-stage framework. The first stage was a retrospective case-control study, and the second stage was a prospective interventional study. Both phases strictly adhered to the STROBE statement recommendations to ensure transparency and reproducibility in case selection, variable definition, and statistical analysis.

In the case-control study phase, a total of 535 children with a definitive diagnosis of AD in the Department of Pediatrics of our hospital between 2022 and 2024 were enrolled as the case group. Data for the case group were extracted from the electronic medical record system, including allergen-specific IgE antibody test results, environmental monitoring indicators, and Staphylococcus aureus culture results from skin microbiota testing reports. The control group was matched 1:1 by age (within a range of ±3 months) and sex, comprising healthy children who underwent health examinations during the same period and were confirmed by questionnaire to have no history of allergic or skin diseases. A total of 535 children were also included in the control group.

**Figure 1:**
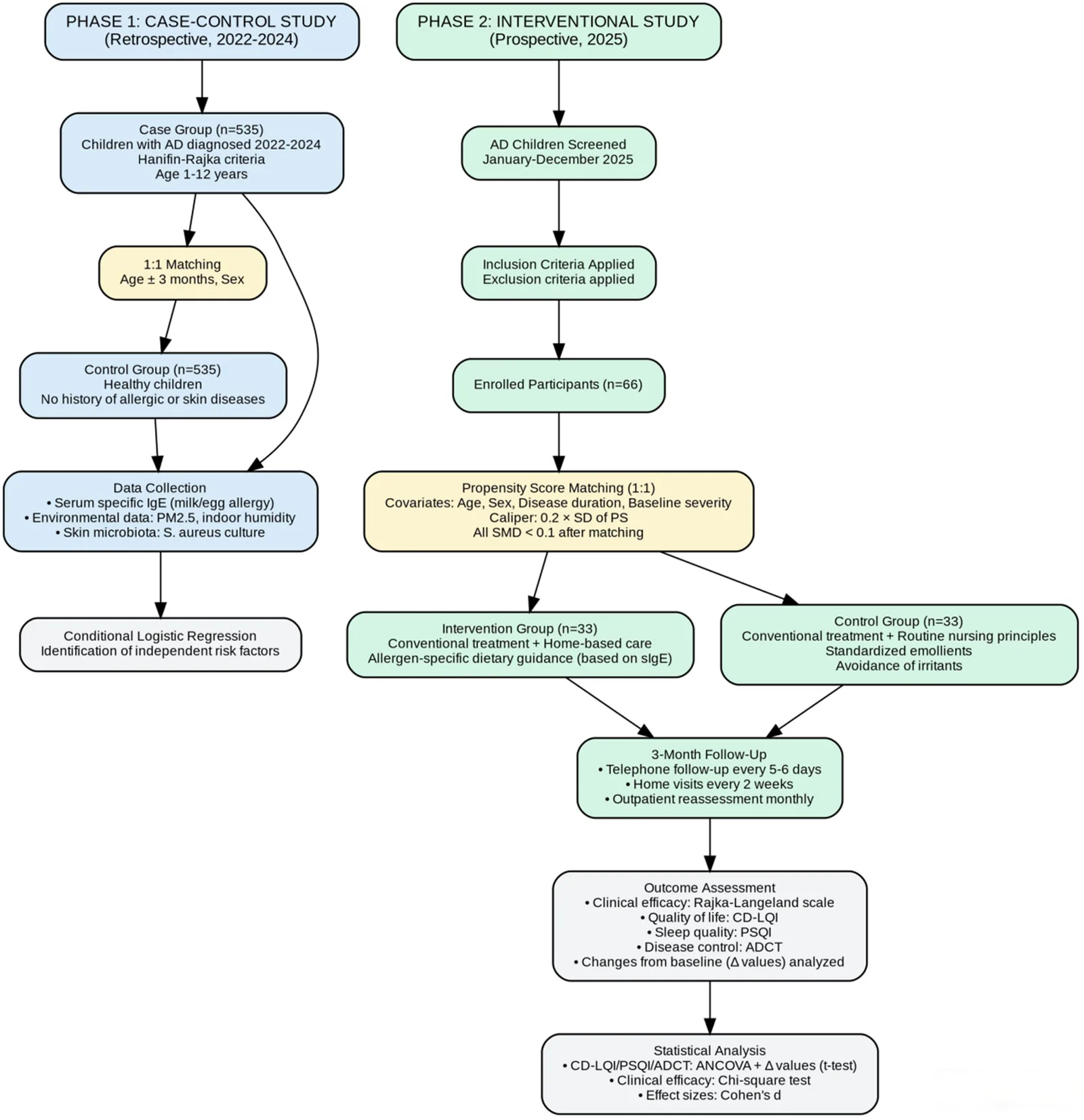
Flowchart of the study design.

In the subsequent interventional study phase, the study population consisted of children with AD who met the full inclusion criteria between January and December 2025. A total of 66 children were ultimately enrolled. To balance baseline characteristics, propensity score matching (PSM) was introduced to further adjust for variables including age, sex, disease duration, and disease severity. Propensity scores were estimated using a logistic regression model incorporating all predefined covariates. Patients were then matched using a 1:1 nearest-neighbor matching algorithm with a caliper width of 0.2 times the standard deviation of the propensity score. After matching, 33 children were retained in each of the intervention and control groups, with standardized mean differences (SMDs) for all covariates controlled below 0.1, indicating successful balance. The overall study workflow is illustrated in Figure 1.

### 2.2 Study participants

Case-control study phase: All pediatric patients met the Hanifin-Rajka diagnostic criteria and were aged between 1 and 12 years. To ensure the reliability of the analytical results, individuals with severe systemic diseases, immunodeficiency, or a history of immunosuppressant or biologic agent use within the preceding three months were excluded.

Interventional study phase: All pediatric patients were clinically diagnosed with atopic dermatitis. Their family members or legal guardians were required to be lucid, possess intact cognitive abilities and basic listening, speaking, reading, and writing skills, have a fixed residential address, and have complete clinical documentation available. Exclusion criteria encompassed the following conditions: presence of severe active infection; concomitant severe functional impairment or complications involving vital organs such as the heart, liver, lungs, or kidneys; diagnosis of immunodeficiency or other immune-related disorders; and the presence of psychiatric disorders or significant cognitive-communication barriers in the guardian.

### 2.3 Data collection for environmental and dietary factors

Environmental exposure data were collected as follows:

PM2.5 exposure: Daily mean PM2.5 concentration data were obtained from the local Ecological Environment Bureau's urban air quality monitoring stations. For each participant, the average PM2.5 concentration during the winter season (November to February) of the year of diagnosis (cases) or health examination (controls) was calculated based on the monitoring station nearest to the participant's residential district.

Indoor humidity: Indoor relative humidity data were recorded by participating families using commercially available hygrometers placed in the child's primary living space (bedroom). Hygrometers were positioned at a height of 1.0–1.5 m above floor level, away from windows, doors, heat sources, and humidifiers/dehumidifiers. All hygrometers were calibrated prior to use using the saturated salt solution method (sodium chloride, 75% relative humidity) according to the manufacturer's instructions, with a specified accuracy of ±5% relative humidity. Families were instructed to record humidity readings twice daily (07:00–08:00 and 20:00–21:00) throughout the winter season (November to February). Monthly mean values were calculated, and the seasonal mean value was derived from these monthly averages for analysis. Families were also asked to record daily ventilation duration (open windows) and the use of humidifiers or dehumidifiers in a simple log; however, due to the retrospective nature of this phase, these behavioral factors could not be objectively quantified and were therefore not included in the primary analysis. These data represent objective instrumental measurements rather than subjective patient recall.

Dietary allergen data: Early milk or egg sensitization was defined based on serum specific IgE testing (ImmunoCAP system, Thermo Fisher Scientific) with a cutoff of ≥0.35 kU/L. It is important to note that specific IgE positivity indicates sensitization rather than clinical food allergy; the diagnosis of clinical food allergy would require confirmation by oral food challenge, which was not performed in this retrospective cohort (8). These are clinical diagnostic data obtained from electronic medical records.

Microbial data: Staphylococcus aureus colonization status was assessed based on skin swab culture results obtained from the lesional skin of cases and from corresponding skin sites of controls. Swabs were taken from the right nostril, five flexural sites (anterior neck, antecubital fossae and popliteal fossae), and the skin area most severely affected, with preference for oozing or crusting lesions where present (9). At each site, a sterile swab was rotated over a skin area of approximately 4 cm^2^ for approximately five seconds. The targeted lesion phenotypes included eczematous lesions with oozing, crusting, erythema, or scaling (9). A colonization rate >50% was defined as the proportion of culture-positive samples exceeding 50% of the total samples in each group.

### 2.4 Intervention methods

In terms of intervention measures, both groups received conventional treatment. The “conventional treatment” comprised: (1) basic skin care with regular use of emollients/moisturizers; (2) topical corticosteroids of appropriate potency according to disease severity (hydrocortisone 1% for mild disease, or medium-potency agents such as betamethasone valerate 0.1% for moderate disease); (3) oral antihistamines (cetirizine or loratadine) as needed for pruritus control. No biologic agents (e.g., dupilumab, omalizumab) or systemic immunosuppressants were used in either group during the study period (10). The control group followed routine nursing principles, which primarily encompassed guidance on the standardized use of emollients and recommendations for avoiding irritants.

The intervention group, on this basis, implemented a multidimensional management protocol of “home-based care combined with allergen-specific dietary guidance.” This specifically included nutritional status assessment and individualized allergen avoidance guidance conducted by professionals. Crucially, dietary modifications were implemented only for children with documented sensitization to specific foods based on serum specific IgE testing (≥0.35 kU/L). Dietary plans were formulated according to the child's disease condition, dietary habits, and nutritional requirements to ensure balanced daily nutrition, enhance immunity and resistance, and avoid the consumption of spicy and irritating foods. The intake of fresh vegetables and fruits, as well as foods rich in calories and high-quality protein, was encouraged. For children with confirmed milk and/or egg sensitization, strict avoidance of the offending allergens and their derived products was recommended. For children without documented food sensitization, no empirical food avoidance was recommended, in accordance with current clinical guidelines (11). A registered clinical nutritionist conducted dynamic nutritional assessments at enrollment, one month, and three months post-intervention. Assessments included 24 h dietary recalls, monitoring of weight-for-age growth curves, and evaluation of micronutrient intake. For children on milk/egg avoidance diets, appropriate substitutes were recommended, including extensively hydrolyzed formula for infants and alternative protein sources (e.g., meat, fish, legumes) for older children, to prevent growth restriction or micronutrient deficiencies.

Simultaneously, to assist parents in establishing reasonable treatment expectations, healthcare providers systematically conveyed disease-related information at the initial stage of diagnosis and treatment. This was combined with dedicated nursing guidance, standardized technical demonstrations, and regular home visits to gradually enhance parental confidence in the overall treatment strategy. A standardized home care manual was compiled in conjunction with this effort, clearly listing the names, primary effects, and precautions for topical preparations (such as creams and ointments) and oral medications, enabling parents to review and reference the information at any time, thereby effectively improving the standardization of home medication and the quality of care. Regarding residential environment management, it was recommended to maintain indoor temperature within an optimal range of 18–20 °C. During summer, attention was paid to avoiding direct sunlight exposure, activities in public swimming pools, and densely crowded places. For children with confirmed dust mite allergy, comprehensive anti-mite measures were implemented for bedroom bedding, including regular dust removal and ventilation, washing fabrics in hot water above 55 °C (at least once a week), supplemented by sun exposure and beating. Concurrently, appropriate room temperature regulation was employed to inhibit dust mite proliferation at the source. Parents were advised to help children keep their nails trimmed short and to select soft, breathable cotton clothing while minimizing the use of wool, silk, or synthetic fiber products. Bathing was to be performed gently, avoiding excessively hot water, harsh soaps, or antiseptic solutions. Immediately after bathing, a sufficient amount of emollient was to be applied to lock in moisture and alleviate pruritus (12–14).

Furthermore, parents were instructed to carefully observe the child's overall condition, including mental status, rash progression, vesiculation and crusting, changes in lesion color, pruritus severity, and potential accompanying digestive symptoms. When necessary, a simple diary could be used to record disease fluctuations, providing accurate information during communication with follow-up nurses. Throughout the caregiving process, parents were required to maintain ample patience, avoiding expressions of aversion or excessive anxiety in response to changes in the child's skin appearance. Instead, they were encouraged to foster a positive and stable parent-child interaction and therapeutic attitude, thereby creating a favorable psychological environment for the child's recovery. Finally, telephone follow-ups were conducted every 5–6 days, and home visits were performed once every two weeks. During follow-up, rash progression and overall disease outcome were documented in detail, parental mastery of medication use was verified, and medication regimens and health education priorities were dynamically adjusted based on actual circumstances. If the atopic dermatitis score showed no significant improvement within one week, the child was required to attend an outpatient clinic for further evaluation. All children underwent a three-month follow-up period, with outpatient reassessments conducted once monthly.

### 2.5 Outcome measures

In the case-control study phase, dietary factors were defined as early milk or egg sensitization, with a serum specific IgE level ≥0.35 kU/L serving as the diagnostic criterion. Regarding environmental factors, the primary considerations were winter indoor humidity (data collected annually from November to February) based on family hygrometer recordings and the annual average concentration of PM2.5 obtained from local environmental monitoring stations. Microbial factors were assessed based on the positive culture rate of Staphylococcus aureus from skin swabs as an indicator of colonization status.

Efficacy evaluation employed a multidimensional index system encompassing four core dimensions: clinical efficacy, quality of life, sleep quality, and disease control status. Clinical efficacy was determined according to the Rajka and Langeland severity grading scale for atopic dermatitis, which integrates three parameters: the extent of skin involvement, scored as 1 point for <9% of total body surface area, 2 points for 9%–36%, and 3 points for >36%; the duration of lesion remission, scored as 1 point for >3 months, 2 points for <3 months, and 3 points for persistent non-remission; and the degree of pruritus interfering with sleep, scored as 1 point for occasional interference, 2 points for frequent interference, and 3 points for severe interference precluding sleep. The sum of these three scores yields a total score, with 3–4 points classified as mild, 4.5–7.5 points as moderate, and 8–9 points as severe. Assessment of disease change was based on the direction of score shift and crossover between severity grades: a reduction from severe to moderate or from moderate to mild was considered effective; a direct reduction from severe to mild or from mild to clinical remission was considered markedly effective; cases with no decrease in score or no grade crossover were classified as ineffective. Effective and markedly effective cases were combined to calculate the overall response rate in subsequent statistical analyses. Quality of life was assessed using the Children's Dermatology Life Quality Index (CDLQI), which covers ten dimensions including social interactions, family life, and emotional impact. Each item is scored from 0 to 3, with a maximum total score of 30, where higher scores indicate a greater negative impact on quality of life. Sleep quality was evaluated using the Pittsburgh Sleep Quality Index (PSQI), a tool comprising seven assessment dimensions, each scored from 0 to 3, yielding a total score of 21. Higher scores reflect more severe sleep disturbance. Disease control status was measured using the Atopic Dermatitis Control Tool (ADCT), which consists of six questions reflecting symptom fluctuations and disease status over the preceding week. Each question is scored from 0 (none) to 4 (very severe), with a total score of 24. An ADCT total score of ≥7 indicates suboptimal disease control. In dynamic assessment, an increase of ≥5 points from the previous score signifies worsening control, whereas a decrease of ≥5 points indicates symptomatic improvement.

### 2.6 Statistical analysis

For the case-control phase, conditional logistic regression models were employed for analysis to account for the 1:1 matched pairs, incorporating covariates including age, sex, allergy history, and environmental exposure history. Adjusted odds ratios (AOR) and their 95% confidence intervals (CI) were calculated, and the goodness-of-fit of the model was evaluated using the Hosmer–Lemeshow test.

For the interventional phase, propensity score matching (PSM) was performed using a logistic regression model including age, sex, disease duration, and baseline disease severity as covariates. Patients were matched 1:1 using nearest-neighbor matching with a caliper of 0.2 times the standard deviation of the propensity score. Balance between groups after matching was assessed using standardized mean differences (SMDs), with SMD <0.1 considered indicative of good balance.

All data computations were performed using SPSS 28.0 software (IBM Corp., Armonk, NY, USA), and statistical charts were generated and optimized using GraphPad Prism 8 software (GraphPad Software, San Diego, CA, USA). Differences in the distribution of clinical efficacy between groups were examined using the chi-square test. For continuous outcomes (CD-LQI, PSQI, and ADCT scores), analysis of covariance (ANCOVA) was performed with baseline scores as covariates and post-treatment scores as dependent variables to adjust for potential baseline differences. As a supplementary analysis, changes in scores from baseline to three months (Δ values) were calculated for each participant, and between-group differences in Δ values were compared using independent samples *t*-test. The two analytical approaches yielded consistent results. A *P*-value <0.05 was considered indicative of a statistically significant difference between groups.

## 3 Results

### 3.1 Baseline characteristics of the case-control cohort

Table 1 presents the baseline characteristics of the case-control cohort (*n* = 1,070). Among the 535 AD cases, the mean age was 6.4 ± 3.1 years, with a male-to-female ratio of 1.12:1. The prevalence of early milk/egg sensitization (sIgE ≥0.35 kU/L) was significantly higher in cases than in controls (38.5% vs. 12.0%). Similarly, cases had a substantially higher proportion of winter indoor humidity <40% (45.6% vs. 29.2%), PM2.5 >35 μg/m^3^ (52.0% vs. 30.1%), and S. aureus colonization rate >50% (48.6% vs. 17.2%) compared with controls.

**Table 1:** Baseline characteristics of the case-control cohort.

Characteristic	Cases (*n* = 535)	Controls (*n* = 535)
Age, years, mean ± SD	6.4 ± 3.1	6.4 ± 3.0
Male, *n* (%)	282 (52.7)	282 (52.7)
Early milk/egg sensitization (sIgE ≥0.35 kU/L), *n* (%)	206 (38.5)	64 (12.0)
Winter indoor humidity <40%, *n* (%)	244 (45.6)	156 (29.2)
PM2.5 >35 μg/m^3^, *n* (%)	278 (52.0)	161 (30.1)
S. aureus colonization rate >50%, *n* (%)	260 (48.6)	92 (17.2)

### 3.2 Conditional logistic regression analysis

The results of conditional logistic regression analysis demonstrated that early milk or egg sensitization, low winter indoor relative humidity, high exposure to atmospheric fine particulate matter (PM2.5), and excessive colonization of Staphylococcus aureus on the skin were all independently and significantly associated with an increased risk of pediatric atopic dermatitis (all *P* < 0.001; see Table 2 for details). Among these factors, a Staphylococcus aureus colonization rate exceeding 50% was associated with the most pronounced elevation in risk (AOR = 4.08), followed in descending order by early food sensitization (AOR = 3.18), PM2.5 exposure (AOR = 2.48), and winter indoor humidity below 40% (AOR = 1.92).

**Table 2:** Conditional logistic regression analysis of risk factors for AD onset.

Risk factor	AOR	95% CI	*P*
Early milk/egg sensitization	3.18	2.08–4.78	<0.001
Indoor humidity in winter <40%	1.92	1.30–2.71	<0.001
PM2.5 >35 μg/m^3^	2.48	1.80–3.50	<0.001
Staphylococcus aureus colonization rate >50%	4.08	2.98–5.58	<0.001

### 3.3 Interventional study

#### 3.3.1 Baseline characteristics

After propensity score matching, the intervention group comprised 33 cases, with a male-to-female ratio of 18:15, an age range of 4–16 years, and a mean age of (8.94 ± 2. 18) years. The disease duration ranged from 0.5 to 1.8 years, with a mean duration of (0.99 ± 0.51) years. The mean body weight was (26.78 ± 1.83) kg. Regarding parental education level, 17 cases had completed high school education or above, and 16 cases had completed middle school education or below. Twenty-nine cases resided in urban areas, and 4 cases resided in rural areas. The control group comprised 33 cases, with a male-to-female ratio of 19:14, an age range of 4–16 years, and a mean age of (9.03 ± 2.25) years. The disease duration ranged from 0.5 to 1.8 years, with a mean duration of (1.01 ± 0.42) years. The mean body weight was (26.83 ± 1.65) kg. Regarding parental education level, 20 cases had completed high school education or above, and 13 cases had completed middle school education or below. Thirty cases resided in urban areas, and 3 cases resided in rural areas. After PSM, all covariates demonstrated good balance with SMD values below 0.1, indicating comparability between the groups (Table 3).

**Table 3:** Comparison of baseline characteristics between the two groups.

Characteristic	Intervention group (*n* = 33)	Control group (*n* = 33)	SMD
Gender ratio (male:female)	0.760416667	0.801388889	0.035
Age (years), mean ± SD	8.94 ± 2.18	9.03 ± 2.25	0.041
Disease duration (years), mean ± SD	0.99 ± 0.51	1.01 ± 0.42	0.043
Body mass (kg), mean ± SD	26.78 ± 1.83	26.83 ± 1.65	0.029
Parental education (high school and above), *n*	17	20	0.068
Place of residence (urban), *n*	29	30	0.039

SMD, standardized mean difference; SMD <0.1 indicates good balance between groups.

#### 3.3.2 Efficacy analysis

**Figure 2:**
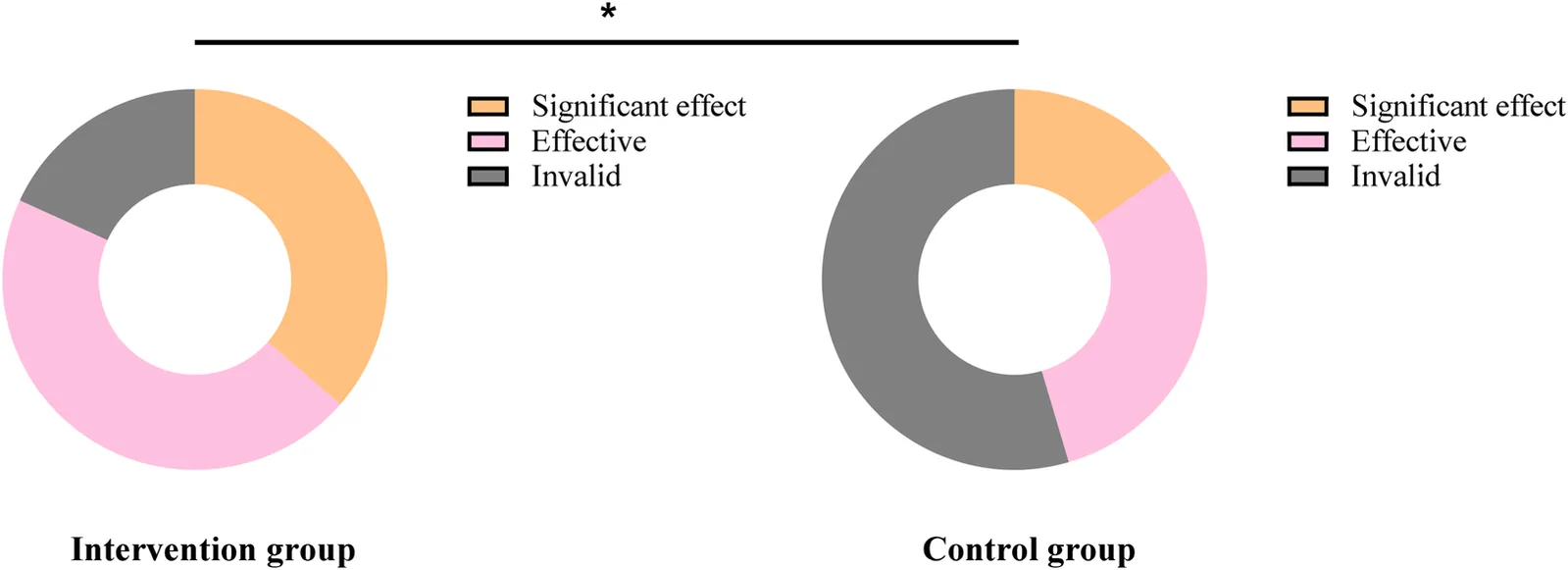
Comparison of clinical efficacy between the two groups. * Indicates a statistically significant difference between the two groups, *P* < 0.05.

The clinical efficacy rate in the intervention group was significantly higher than that in the control group (81.82% vs. 45.45%), and the difference was statistically significant (*P* < 0.05), as shown in Figure 2.

#### 3.3.3 Quality of life

**Figure 3:**
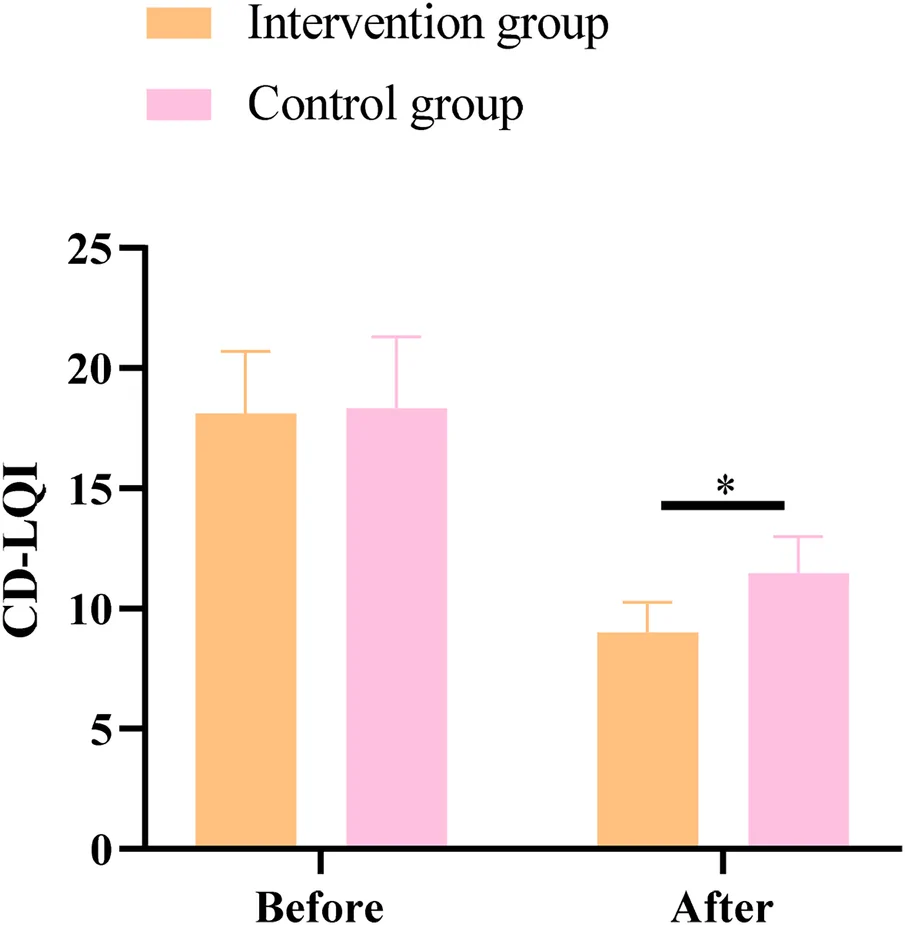
Comparison of CD-LQI scores before and after treatment between the two groups. * Indicates a statistically significant difference between the two groups, *P* < 0.05.

Before treatment, there was no statistically significant difference in CD-LQI scores between the two groups (intervention group: 18.11 ± 2.59 vs. control group: 18.32 ± 2.98, *P* = 0.758). After three months of intervention, the CD-LQI score in the intervention group (9.01 ± 1.24) was significantly lower than that in the control group (11.47 ± 1.52) (*P* < 0.001). After adjustment for baseline scores using ANCOVA, the intervention group demonstrated significantly lower post-treatment CD-LQI scores than the control group (adjusted mean difference: 2.46, 95% CI: 1.72–3.20, *P* < 0.001). The mean reduction (Δ) in CD-LQI score from baseline to three months was also significantly greater in the intervention group (9.10 ± 2.01) than in the control group (6.85 ± 2.21), with a statistically significant difference (*P* < 0.001), indicating that the multidimensional intervention produced substantially greater improvement in quality of life than conventional care. The effect size (Cohen's *d*) for the between-group difference in CD-LQI improvement was 1.07, indicating a large effect (Figure 3).

Supplementary Table 1 presents the changes in the three sub-items of the Rajka-Langeland scale from baseline to three months. The most pronounced improvement was observed in pruritus severity (intervention group: from 2.52 ± 0.51 to 1.21 ± 0.42; control group: from 2.48 ± 0.51 to 1.70 ± 0.47), followed by skin involvement extent and remission duration.

Supplementary Table 2 presents the domain-specific sub-item analyses for the CDLQI, PSQI, and ADCT scales. For the CDLQI, the most substantial improvements in the intervention group were observed in the domains of “symptoms and feelings” (itching, sleep) and “leisure” (sports/social activities). For the PSQI, the “sleep latency” and “sleep efficiency” components showed the greatest improvement. For the ADCT, the items assessing “overall symptom severity” and “frequency of itching” demonstrated the largest reductions.

#### 3.3.4 Sleep status

**Figure 4:**
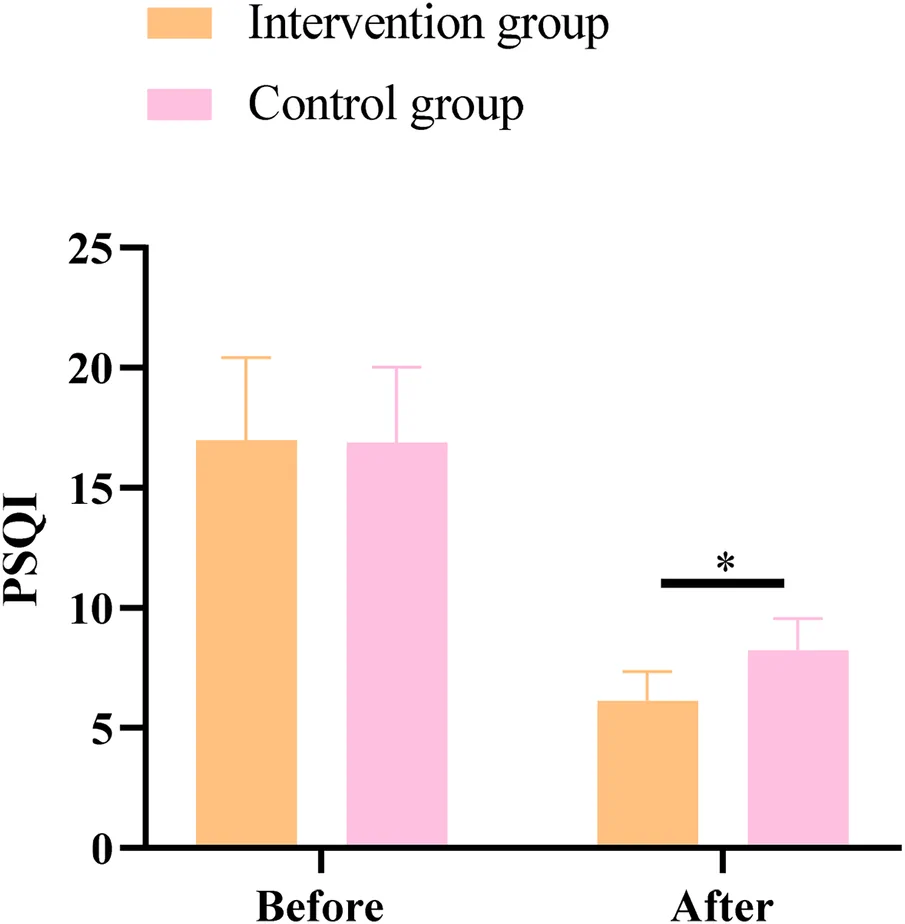
Comparison of PSQI scores before and after treatment between the two groups. * Indicates a statistically significant difference between the two groups, *P* < 0.05.

Before treatment, there was no statistically significant difference in PSQI scores between the two groups (intervention group: 16.98 ± 3.45 vs. control group: 16.89 ± 3.15, *P* = 0.912). After three months of intervention, the PSQI score in the intervention group (6.14 ± 1.21) was significantly lower than that in the control group (8.23 ± 1.33) (*P* < 0.001). After adjustment for baseline scores using ANCOVA, the intervention group demonstrated significantly lower post-treatment PSQI scores than the control group (adjusted mean difference: 2.09, 95% CI: 1.45–2.73, *P* < 0.001). The mean reduction (Δ) in PSQI score from baseline to three months was significantly greater in the intervention group (10.84 ± 3.12) than in the control group (8.66 ± 2.78), with a statistically significant difference (*P* < 0.001), demonstrating that the multidimensional intervention more effectively improved sleep quality than conventional care. The effect size (Cohen's *d*) for the between-group difference in PSQI improvement was 0.74, indicating a moderate-to-large effect (Figure 4).

#### 3.3.5 Disease control

**Figure 5:**
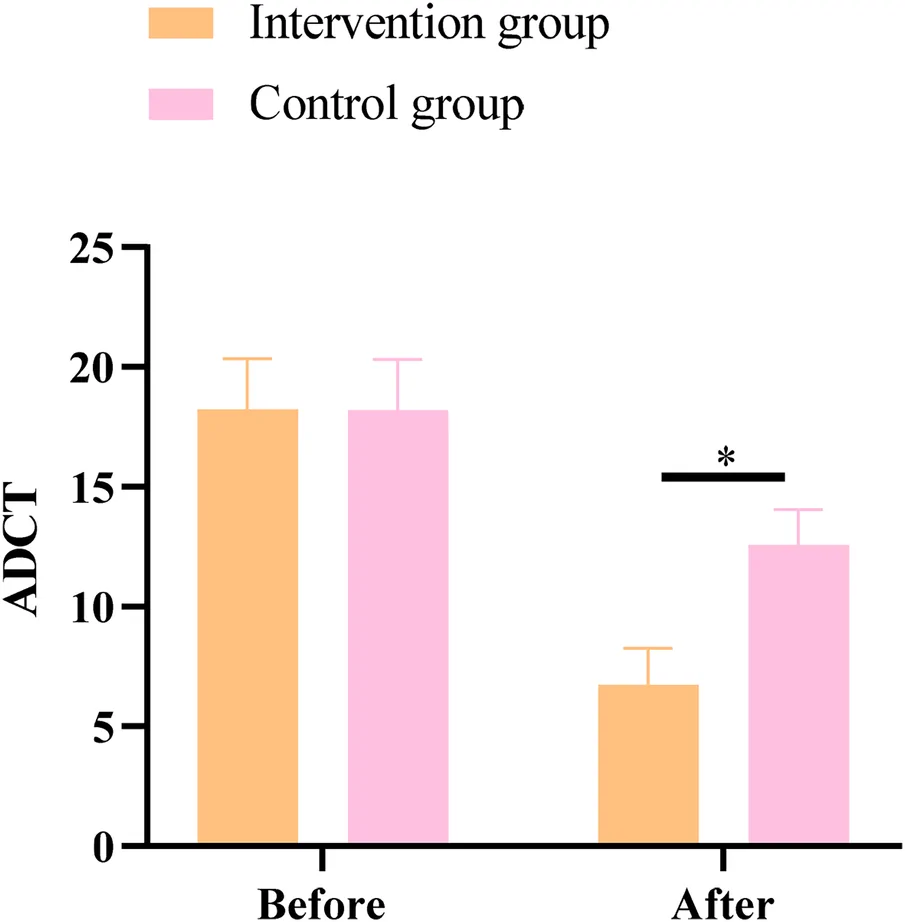
Comparison of ADCT scores before and after treatment between the two groups. * Indicates a statistically significant difference between the two groups, *P* < 0.05.

Before treatment, there was no statistically significant difference in ADCT scores between the two groups (intervention group: 18.23 ± 2.11 vs. control group: 18.19 ± 2.13, *P* = 0.938). After three months of intervention, the ADCT score in the intervention group (6.73 ± 1.54) was significantly lower than that in the control group (12.58 ± 1.48) (*P* < 0.001). After adjustment for baseline scores using ANCOVA, the intervention group demonstrated significantly lower post-treatment ADCT scores than the control group (adjusted mean difference: 5.85, 95% CI: 5.08–6.62, *P* < 0.001). The mean reduction (Δ) in ADCT score from baseline to three months was significantly greater in the intervention group (11.50 ± 2.34) than in the control group (5.61 ± 2.21), with a statistically significant difference (*P* < 0.001), indicating that the multidimensional intervention achieved substantially better disease control than conventional care. The effect size (Cohen's *d*) for the between-group difference in ADCT improvement was 2.57, indicating a very large effect (Figure 5).

## 4 Discussion

This study systematically examined the interactive effects of dietary and environmental factors in the management of AD through a multidimensional intervention strategy. The findings confirm that early milk/egg sensitization, low winter indoor humidity, elevated PM2.5 exposure, and excessive S. aureus colonization are independently associated with increased odds of pediatric AD onset. More importantly, the prospective interventional trial demonstrated that a comprehensive home-based care protocol combined with allergen-specific dietary guidance achieved superior clinical outcomes compared with conventional treatment across multiple domains, including overall efficacy, quality of life, sleep quality, and disease control.

### 4.1 Specific triggering mechanisms of individual risk factors

The association between early milk/egg sensitization and AD onset has been consistently observed in both clinical practice and epidemiological studies. In sensitized children, milk proteins such as casein and egg proteins such as ovalbumin induce B cells to produce specific IgE antibodies that bind to Fc*ε*RI receptors on skin mast cells (15). Upon re-exposure, antigen cross-linking triggers mast cell degranulation and the release of histamine, leukotrienes, and prostaglandins, producing pruritus, erythema, and edema. Beyond this immediate immune response, persistent allergen exposure also disrupts keratinocyte terminal differentiation and compromises the epidermal lipid bilayer (16, 17). Earlier work has shown that TEWL values in non-lesional skin of children with food allergy-associated AD are substantially higher than those in healthy children, suggesting that barrier impairment exists even in rash-free areas. This creates a self-perpetuating cycle of allergic inflammation and barrier dysfunction (18–20). In our case-control analysis, early milk/egg sensitization was independently associated with a more than threefold increase in the odds of AD. This magnitude of association is consistent with systematic review findings that dietary avoidance can meaningfully reduce disease activity in children with established sensitization. That said, two important clinical caveats warrant emphasis. Current guidelines, including the 2023 AAAAI/ACAAI recommendations, advise against routine food allergy panel testing and empiric elimination diets in unselected AD populations; testing should be reserved for children with clear immediate reactions or persistent disease despite optimized topical therapy (11). Equally important, specific IgE positivity indicates sensitization, not necessarily clinical food allergy; the latter requires confirmation by oral food challenge (9).

Low winter indoor humidity represents a distinct environmental trigger for AD. During heating seasons in northern regions, indoor relative humidity in homes without humidifiers frequently drops below 35%, and clinicians often observe that affected children develop visible scaling and fissuring on extensor surfaces and cheeks under such conditions. The mechanistic link between low humidity and barrier disruption has been traced to filaggrin degradation. Filaggrin is a critical structural protein of the stratum corneum, and its degradation products constitute natural moisturizing factor (NMF), which maintains epidermal hydration. Under dry conditions, increased filaggrin deimination accelerates its breakdown, depleting NMF and compromising barrier integrity (21). Experimental evidence from reconstructed human epidermal models has shown that reducing ambient humidity from 60% to 30% increases TEWL substantially within hours (21). In our study, winter indoor humidity below 40% was associated with a nearly twofold increase in AD odds, an effect likely mediated through downregulation of filaggrin and loricrin as well as functional impairment of tight junction proteins such as claudin-1 (22, 23). These changes increase paracellular permeability and facilitate allergen penetration.

Exposure to PM2.5 at concentrations above 35 μg/m^3^ has been increasingly recognized as a contributor to AD pathogenesis. The fine particles can penetrate the superficial skin barrier and act directly on keratinocytes and local immune cells. Mechanistically, PM2.5 exposure generates excessive reactive oxygen species (ROS) and activates AhR and NF-*κ*B signaling pathways (24). This oxidative stress cascade, operating through TNF-*α*-mediated and AhR-dependent mechanisms, reduces expression of filaggrin, loricrin, keratin-1, and corneodesmosin—all essential for epidermal integrity. The resulting barrier compromise increases TEWL and allergen permeability (24). There is also evidence that PM2.5-induced barrier dysfunction promotes cutaneous dysbiosis, favoring S. aureus colonization while reducing beneficial commensals such as S. epidermidis, which in turn activates dendritic cells and drives Th2-polarized immune responses (24). Meta-analyses (25) have consistently demonstrated a positive association between PM2.5 exposure and the risk of childhood atopic dermatitis. Consistent with these findings, high PM2.5 exposure in our cohort was independently associated with increased odds of AD. Mechanistically, PM2.5 may impair epidermal barrier integrity through oxidative stress and AhR/NF-*κ*B signaling while also promoting Th2-predominant immune responses via the lung–skin axis, thereby facilitating allergic inflammation (26).

Among the risk factors examined, excessive S. aureus colonization showed the strongest association with AD onset. When colonization exceeds a certain threshold, secreted exotoxins—including *δ*-toxin and *α*-toxin—directly damage keratinocytes and immune cells (27). In children with AD, the skin microbiome shows reduced diversity, with S. aureus detected in the vast majority of cases compared with a very small fraction of healthy controls, and colonization density correlates positively with disease severity. In the present study, high colonization was associated with a fourfold increase in AD odds, making it the most potent individual risk factor identified. S. aureus further exacerbates dysbiosis by inhibiting beneficial commensals through biofilm formation, highlighting the importance of strategies aimed at restoring microbial homeostasis.

### 4.2 Common pathogenic pathways: barrier impairment and inflammatory amplification

Despite their distinct initiating events, the risk factors examined in this study converge on two shared downstream pathways: structural disruption of the skin barrier and amplification of local immune inflammation. Whether triggered by allergen-induced differentiation defects, humidity-driven NMF depletion, PM2.5-mediated oxidative injury, or bacterial toxin activity, the net effect is elevated TEWL and reduced stratum corneum hydration. This barrier compromise, even when subclinical, lowers the threshold for irritation and allergen penetration, establishing a feedback loop of progressive impairment. At the inflammatory level, these factors operate through different signaling routes—IgE-Fc*ε*RI, p38 MAPK, NF-*κ*B, and TLR pathways—but ultimately converge on the release of pro-inflammatory cytokines (IL-1*β*, IL-6, TNF-*α*, IL-8) and reinforcement of Th2-type immunity (28–30). This convergence of multiple triggers on common effector pathways explains both the heterogeneity of AD presentations and the limited efficacy of single-modality interventions.

### 4.3 Clinical efficacy of multidimensional intervention and mechanistic validation

The prospective interventional data support the clinical utility of the multidimensional approach. The overall response rate in the intervention group was substantially higher than in the control group, and this difference was reflected across all secondary outcome measures. In the intervention group, children achieved greater improvements in quality of life, sleep quality, and disease control, with effect sizes ranging from moderate to very large depending on the outcome domain. This consistent pattern of benefit across multiple patient-reported and clinician-assessed endpoints suggests that the intervention produced clinically meaningful change that extended beyond symptom reduction to functional and psychosocial domains.

The superiority of the multidimensional protocol can be understood through its simultaneous targeting of multiple nodes in the pathogenic cascade. Dietary management—applied selectively to children with documented sensitization—reduces ongoing allergen-driven immune stimulation. Environmental interventions, including humidity optimization and reduced PM2.5 exposure, attenuate exogenous triggers at their source. Standardized skin care directly supports stratum corneum hydration and physical barrier repair. Combined, these measures are likely to have a synergistic effect, interrupting the “barrier impairment—inflammatory amplification” cycle at several points simultaneously (31–34). This synergistic blockade, rather than the effect of any single component, probably accounts for the observed efficacy advantage over conventional care.

### 4.4 Study limitations and future directions

Several limitations of this study should be considered when interpreting the findings. The retrospective case-control design, while useful for hypothesis generation, is subject to selection and information biases that cannot be fully eliminated despite the use of conditional logistic regression and propensity score matching. Prospective cohort studies would provide stronger evidence for causal inference. The interventional sample size was modest and the follow-up period limited to three months; larger, longer-term studies are needed to assess durability of response and safety. Environmental exposure assessment relied on regional monitoring data and family-recorded hygrometer readings rather than personal portable monitors, which may have introduced exposure misclassification. Furthermore, indoor humidity is influenced by household behaviors such as ventilation practices and the use of humidifiers or dehumidifiers, which were not objectively captured in this retrospective design. Future work should consider individual-level exposure monitoring to improve accuracy. The multi-component nature of the intervention is both a strength and a limitation: while it reflects real-world clinical practice, it precludes isolation of the independent effect of any single component. The observed improvements cannot be attributed to dietary control alone, and this causal overlap must be acknowledged as a fundamental constraint of the study design. Genetic and epigenetic mechanisms were not explored, and future studies incorporating multi-omics approaches could offer deeper insights into individualized treatment pathways. Finally, the classification of food allergy in the retrospective phase was based on serum specific IgE levels rather than oral food challenges, the reference standard for clinical diagnosis. This may have resulted in inclusion of children with asymptomatic sensitization rather than true clinical allergy, and we have accordingly used the term “sensitization” throughout to reflect this distinction.

## 5 Conclusion

In summary, this study provides systematic evidence that early milk/egg sensitization, low indoor humidity, elevated PM2.5 exposure, and heavy S. aureus colonization are independently associated with increased odds of pediatric AD. A multidimensional home-based care protocol combined with sensitization-directed dietary guidance achieved significantly better clinical outcomes than conventional treatment across multiple domains. Dietary modification should be reserved for children with documented sensitization based on specific IgE testing, as empiric elimination in unselected populations is not supported by current guidelines. It bears repeating that specific IgE positivity indicates sensitization, not clinical food allergy; the latter requires oral food challenge confirmation. Future work should incorporate oral food challenges to distinguish sensitization from true allergy and further investigate the molecular underpinnings of AD to enable precision medicine approaches that improve long-term outcomes for affected children.

## Data Availability

The original contributions presented in the study are included in the article/Supplementary Material, further inquiries can be directed to the corresponding author/s.
