# Supplementary material for: The role and management of dietary and environmental factors in pediatric atopic dermatitis
Source: Front Pediatr. 2026 Sep 7;14:1862285. doi: 10.3389/fped.2026.1862285 (PMC13590424; doi:10.3389/fped.2026.1862285)
Supplement: Supplementary file 1 [file Table1.docx]

Supplementary Table 1 presents the changes in the three sub-items of the Rajka-Langeland scale from baseline to three months. The most pronounced improvement was observed in pruritus severity (intervention group: from 2.52±0.51 to 1.21±0.42; control group: from 2.48±0.51 to 1.70±0.47), followed by skin involvement extent and remission duration.

Supplementary Table 1. Changes in Rajka-Langeland Sub-items from Baseline to 3 Months

| Sub-item | Intervention Group (n=33) | | | Control Group (n=33) | | | P for Δ |
| --- | --- | --- | --- | --- | --- | --- | --- |
|  | Baseline | 3 months | Δ | Baseline | 3 months | Δ |  |
| Skin involvement extent (0–3) | 2.21±0.65 | 1.33±0.48 | 0.88±0.56 | 2.18±0.64 | 1.70±0.53 | 0.48±0.51 | <0.01 |
| Remission duration (0–3) | 2.06±0.66 | 1.24±0.44 | 0.82±0.53 | 2.03±0.68 | 1.58±0.50 | 0.45±0.49 | <0.01 |
| Pruritus severity (0–3) | 2.52±0.51 | 1.21±0.42 | 1.31±0.48 | 2.48±0.51 | 1.70±0.47 | 0.78±0.50 | <0.001 |

Supplementary Table 2 presents the domain-specific sub-item analyses for the CDLQI, PSQI, and ADCT scales. For the CDLQI, the most substantial improvements in the intervention group were observed in the domains of "symptoms and feelings" (itching, sleep) and "leisure" (sports/social activities). For the PSQI, the "sleep latency" and "sleep efficiency" components showed the greatest improvement. For the ADCT, the items assessing "overall symptom severity" and "frequency of itching" demonstrated the largest reductions.

Supplementary Table 2. Domain-Specific Sub-item Changes in CDLQI, PSQI, and ADCT

| Scale / Domain | Intervention Group (n=33) Δ | Control Group (n=33) Δ | P |
| --- | --- | --- | --- |
| CDLQI |  |  |  |
| Symptoms and feelings (items 1–2) | 3.42±0.98 | 2.15±0.87 | <0.001 |
| Leisure (items 3–4) | 2.15±0.76 | 1.52±0.69 | <0.01 |
| School/holidays (item 5) | 1.06±0.56 | 0.85±0.51 | 0.12 |
| Personal relationships (items 6–7) | 1.52±0.67 | 1.21±0.58 | 0.05 |
| Sleep (item 8) | 0.85±0.51 | 0.64±0.49 | 0.09 |
| Treatment (items 9–10) | 0.91±0.52 | 0.70±0.47 | 0.08 |
| PSQI |  |  |  |
| Subjective sleep quality | 1.52±0.62 | 1.21±0.55 | 0.04 |
| Sleep latency | 1.85±0.71 | 1.39±0.61 | <0.01 |
| Sleep duration | 1.42±0.61 | 1.18±0.56 | 0.10 |
| Habitual sleep efficiency | 1.79±0.67 | 1.33±0.58 | <0.01 |
| Sleep disturbances | 1.33±0.54 | 1.06±0.50 | 0.04 |
| Use of sleeping medication | 0.48±0.51 | 0.42±0.50 | 0.63 |
| Daytime dysfunction | 1.64±0.60 | 1.33±0.54 | 0.03 |
| ADCT |  |  |  |
| Overall symptom severity | 2.24±0.75 | 1.12±0.60 | <0.001 |
| Frequency of itching | 2.06±0.70 | 1.03±0.58 | <0.001 |
| Intensity of itching | 1.97±0.68 | 0.97±0.55 | <0.001 |
| Interference with sleep | 1.76±0.64 | 0.85±0.51 | <0.001 |
| Interference with daily life | 1.67±0.60 | 0.82±0.50 | <0.001 |
| Impact on mood/emotions | 1.61±0.58 | 0.79±0.49 | <0.001 |
